# 2013 Trends in anogenital warts incidence: Potential impact of human papillomavirus vaccination, TennCare 2006–2015

**DOI:** 10.1017/cts.2018.308

**Published:** 2018-11-21

**Authors:** Jaimie Z. Shing, Marie Griffin, Manideepthi Pemmaraju, Edward Mitchel, Pamela Hull

**Affiliations:** Vanderbilt University Medical Center

## Abstract

OBJECTIVES/SPECIFIC AIMS: We aimed to assess trends in incidence of genital warts across human papillomavirus (HPV) vaccine-eligible and nonvaccine-eligible age groups to determine the impact of the HPV vaccine among Medicaid enrollees in the state of Tennessee. METHODS/STUDY POPULATION: We analyzed 2006–2014 medical and pharmaceutical claims data from TennCare (Tennessee’s Medicaid program) enrollees aged 15–64 years. Incident cases of genital warts were defined as persons 12 months disease free and: (1) a diagnosis of condyloma acuminatum, or (2) a diagnosis of viral warts and genital-specific procedure, or (3) a prescription for genital warts medication and genital-specific procedure. Mann-Kendall trend tests were performed to assess for significant trends in incidence of genital warts by sex and age group; average annual percent changes were calculated to quantify these trends. RESULTS/ANTICIPATED RESULTS: Our analysis is in progress. We hypothesize that we will observe declines in genital warts among younger, vaccine-eligible age groups and no changes in older, nonvaccine-eligible age groups, with largest declines among females aged 15–19 years from 2006 to 2014. We also expect to see declines among younger males due to herd protection, with greater declines after 2011, when the vaccine was approved for males. DISCUSSION/SIGNIFICANCE OF IMPACT: Significant declines among younger compared with older age groups would suggest HPV vaccine effectiveness for preventing genital warts.

